# Light protection of parenteral nutrition, cholestasis, and other prematurity-related morbidities in premature infants

**DOI:** 10.3389/fped.2022.900068

**Published:** 2022-08-03

**Authors:** Christie Clauss, Valentyna Tack, Maria Macchiarulo, Meredith Akerman, Gladys El-Chaar, Nazeeh Hanna, Caterina Tiozzo

**Affiliations:** ^1^Department of Pharmacy, NYU Langone Hospital–Long Island, Mineola, NY, United States; ^2^Department of Neonatology, Children's National Hospital, Washington, DC, United States; ^3^Department of Pharmacy, Hassenfeld Children's Hospital at NYU Langone, New York, NY, United States; ^4^Department of Foundations of Medicine, NYU Langone Hospital–Long Island, Mineola, NY, United States; ^5^Department of Pharmacy, St. John's University College of Pharmacy and Health Sciences, Queens, NY, United States; ^6^Division of Neonatology, Department of Pediatrics, New York University Long Island School of Medicine, Mineola, NY, United States

**Keywords:** parenteral nutrition, premature infants, light protection, direct hyperbilirubinemia, intestinal failure-associated liver disease, cholestasis, bronchopulmonary dysplasia

## Abstract

**Introduction:**

Parenteral Nutrition (PN) can lead to intestinal failure associated liver disease (IFALD). There are no human studies to date studying specifically the benefits of light-protection on neonatal IFALD. Recently, the European Medicines Agency and the American Society for Parenteral and Enteral Nutrition (ASPEN) both recommended full light protection of PN to reduce the risk of adverse clinical outcomes.

**Objective:**

The primary objective of this study was to evaluate the impact of light-protecting PN on the incidence of cholestasis and peak direct bilirubin levels in premature infants.

**Study design:**

Retrospective chart review of preterm infants requiring PN for a minimum of 2 weeks with or without light-protection. After light protection of the PN solution, primary outcomes (including cholestasis and direct bilirubin levels) of both groups were compared. Secondary outcomes include evaluation of bronchopulmonary dysplasia (BPD), necrotizing enterocolitis (NEC), retinopathy of prematurity (ROP), sepsis and mortality.

**Results:**

A total of 50 preterm infants <37 weeks gestation were included, 25 infants in each group. There was a statistically significant decrease in the rate of cholestasis (12 vs. 3, *p* = 0.005), median peak direct bilirubin levels (1.7 vs. 0.9 mg/dL, p = 0.02) and total bilirubin levels (4.1 vs. 3.4, *p* = 0.05) in the light-protection group compared to no light-protection group. There was a decrease in the incidence of severe BPD (with an increase of mild BPD, resulting in the same overall BPD rate) in the light-protection compared to no light-protection group (7 vs. 15, *p* = 0.0223). There was no difference in NEC, ROP, sepsis or mortality.

**Conclusion:**

Our study supports that the practice of light-protecting PN may reduce the incidence of IFALD in premature infants. Moreover, there was a trend toward decreased incidence of severe BPD in the light-protection group. Further light protection studies are needed to confirm these findings.

## Introduction

Parenteral nutrition (PN) is essential for the care of premature infants who are unable to tolerate enteric nourishment (1). One of the major complications of prolonged PN is the development of hepatic dysfunction, referred to as intestinal failure-associated liver disease (IFALD) or PN-associated cholestasis (PNAC) and defined as a serum direct bilirubin of ≥2 mg/dl (2). The severity ranges from mild to significant hepatic injury and is associated with the duration of PN therapy (3).

The etiology of IFALD remains incompletely understood but suggested contributing factors include the type of lipid emulsion and interruption of enterohepatic circulation (4). It is well established that PN preparations form oxidants when exposed to light and that premature infants lack defense mechanisms due to immature physiology (5). Cell damage from these oxidants can play a role in the development of prematurity-related morbidities such as bronchopulmonary dysplasia (BPD) (6, 7). The available therapies to treat IFALD are limited but include administration of ursodiol and cycling PN with clear fluids (8). Photoprotection of PN was shown to reduce the amount of infused oxidants in premature infants (9). To our knowledge, only animal models have been used to study the hepatobiliary effects of protecting PN products from light. In 2017, Chessex and colleagues published a review paper that made a strong recommendation for human studies addressing the use of photoprotection to decrease the risk of IFALD (7). In 2019, the European Medicines Agency recommended that PN products be light-protected to reduce the risk of adverse clinical outcomes (10). Recently, the American Society for Parenteral and Enteral Nutrition (ASPEN) released a position paper recommending full light protection of PN products (11).

The primary objective of this study was to evaluate the impact of light-protecting PN on the incidence of cholestasis and on the level of direct bilirubin in premature infants. Secondary objectives included evaluation of the incidence of BPD, retinopathy of prematurity (ROP), sepsis, necrotizing enterocolitis (NEC), and death in our population.

## Materials and methods

### Study design

This single-center retrospective chart review evaluated premature infants receiving PN therapy at a Level III Neonatal Intensive Care Unit/Regional Perinatal Center (NICU/RPC) between September 2017 and January 2019. Light protection using amber bags for two-in-one PN was initiated at our hospital in April 2018. Out of 417 evaluated charts, 25 consecutive patients without PN light-protection prior to April 2018 (*n* = 25) were compared to patients with PN light protection after April 2018 (*n* = 25).

### Study population

**Figure 1:**
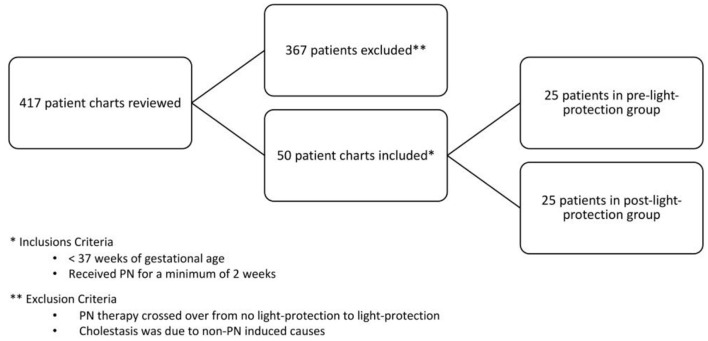
Inclusion and exclusion criteria flow chart.

All preterm infants born <37 weeks gestation, admitted to our NICU, and administered PN for ≥14 days were included in the study. Infants were excluded if they crossed over from no light protection to light protection or if the etiological causes of cholestasis were unrelated to IFALD (Figure 1).

### Data collection and outcomes

All data were collected retrospectively from the review of electronic medical charts, including patient demographics, clinical outcomes, laboratory, and treatment data. The laboratory data were collected weekly (±2 days) while the patient was receiving PN and until off PN for 1 week. If any of these labs were abnormal, they were collected weekly (±2 days) until normalization. The primary outcome of our study was to establish the incidence of cholestasis (defined as a direct bilirubin ≥2 mg/dl) and liver disease. This was achieved by assessing the bilirubin levels (as a marker for cholestasis) and alanine transaminase/aspartate transaminase (ALT/AST) levels (as a marker for liver disease) at birth, at the day of life (DOL) 14 and at the time of the peak direct bilirubin value during PN with or without light protection. Secondary outcomes included the incidence of other prematurity-related morbidities (such as NEC, sepsis, ROP, and BPD) and death. Bronchopulmonary dysplasia was defined and classified according to the physiological definition (12) and the respiratory support at 36 weeks (wks) postmenstrual age (PMA) was recorded as well. We documented the diagnoses of intrauterine growth restriction (IUGR), patent ductus arteriosus (PDA), short bowel syndrome (SBS), NEC, culture-positive sepsis (defined as positive blood, cerebrospinal fluid, or urine culture), and ROP using international classification of diseases (ICD)-10 diagnostic codes. Nutritional data collected included: DOL of onset of enteral feeding tolerance, DOL when the patient reached full enteral feeds (defined as 160 ml/kg/day), type of lipid intake, cycling PN (defined as PN infusing for 20 h per day with dextrose for 4 h), use of ursodiol and duration of PN. Phototherapy use and days on phototherapy were also analyzed.

### Parenteral nutrition practice and light protection process

Our unit's nutrition protocol recommends that immediately following birth, premature infants are administered intravenous nutrition with Starter PN (TrophAmine^®^ B.Braun 3%, dextrose 5 or 10% with calcium gluconate 2.33 mEq, and heparin 125 units per 250 ml) which is not light-protected. On DOL one, infants are started on a customized PN regimen; amino acids (Premasol^®^ Baxter), dextrose, electrolytes, and micronutrients were given with vitamins (Infuvite Pediatric^®^, Baxter). Lipids are initiated on DOL one and infused separately, usually starting at 1 g/kg/day with a daily increase of 1 g/kg/day (up to a maximum dose of 3 g/kg/day). Lipids were supplied as Intralipid^®^ 20% (Fresenius Kabi) or SMOFlipid^®^ 20% (Fresenius Kabi) and were used based on the unit's protocol. If infants were switched from one lipid to another, the patient was categorized based on the lipid they received at the time of the serum peak direct bilirubin. Beginning in April 2018, photoprotection of the PN bag was added as a final step of preparation and was maintained throughout the duration of administration. Tubing and Buretrol^®^ (burette drip chamber) remained exposed to light. Our nursing practice was to allow 2 h of PN solution to be in the drip chamber, which was not light-protected. Lipids were not light-protected. The light exposed group (before April 2018) received PN exposed to ambient light. Enteral feeding was introduced, and its amount increased according to our NICU's standardized protocol. There were no changes to the feeding protocol in our unit during the study period. The components of PN were adjusted and individualized daily according to the infant's serum electrolytes and clinical condition. Ursodiol (10–15 mg/kg every 12 h) was initiated if the infant was tolerating enteral feeds, and PN was cycled if the infant was not tolerating enteral feeds; both measures were implemented if direct serum bilirubin was ≥2 mg/dl. This practice did not differ between pre- and post-light protection. Our study was approved with a waiver of consent by our institution's Institutional Review Board.

## Statistical analysis

The pre- and post-light-protection groups were compared using the chi-square or Fisher's exact tests, as deemed appropriate, for categorical variables, and the Mann–Whitney *U*-test was utilized for continuous data. Descriptive statistics were calculated separately by group (median [25th, 75th percentiles] for continuous variables; and frequency and percent for categorical variables).

Univariate logistic regression models were used for the unadjusted and adjusted analysis of cholestasis. Data are presented as odds ratios (*OR*) with their corresponding 95% confidence intervals (*CI*). Results were considered statistically significant at a *p-*value of <0.05.

Analysis of covariance (ANCOVA) was used to examine the association between group (before and after light protection) and direct bilirubin levels, after adjusting for certain possible confounders. A separate ANCOVA model was adjusted for each of the following: birth weight, duration of PN, DOL of onset of enteral feeding tolerance, DOL of reaching full feeds, presence of NEC, type of lipid, and administration of phototherapy and its duration in days.

The standard assumptions of Gaussian residuals and quality of variance were tested. Since the normality assumption was not met for direct bilirubin, the logarithm transformation was used for this analysis. Results were brought to the original units and reported as geometric means with their corresponding lower and upper confidence limits. All analyses were performed using SAS version 9.4 (SAS Institute Inc., Cary, NC).

## Results

### Study population

**Figure 2:**
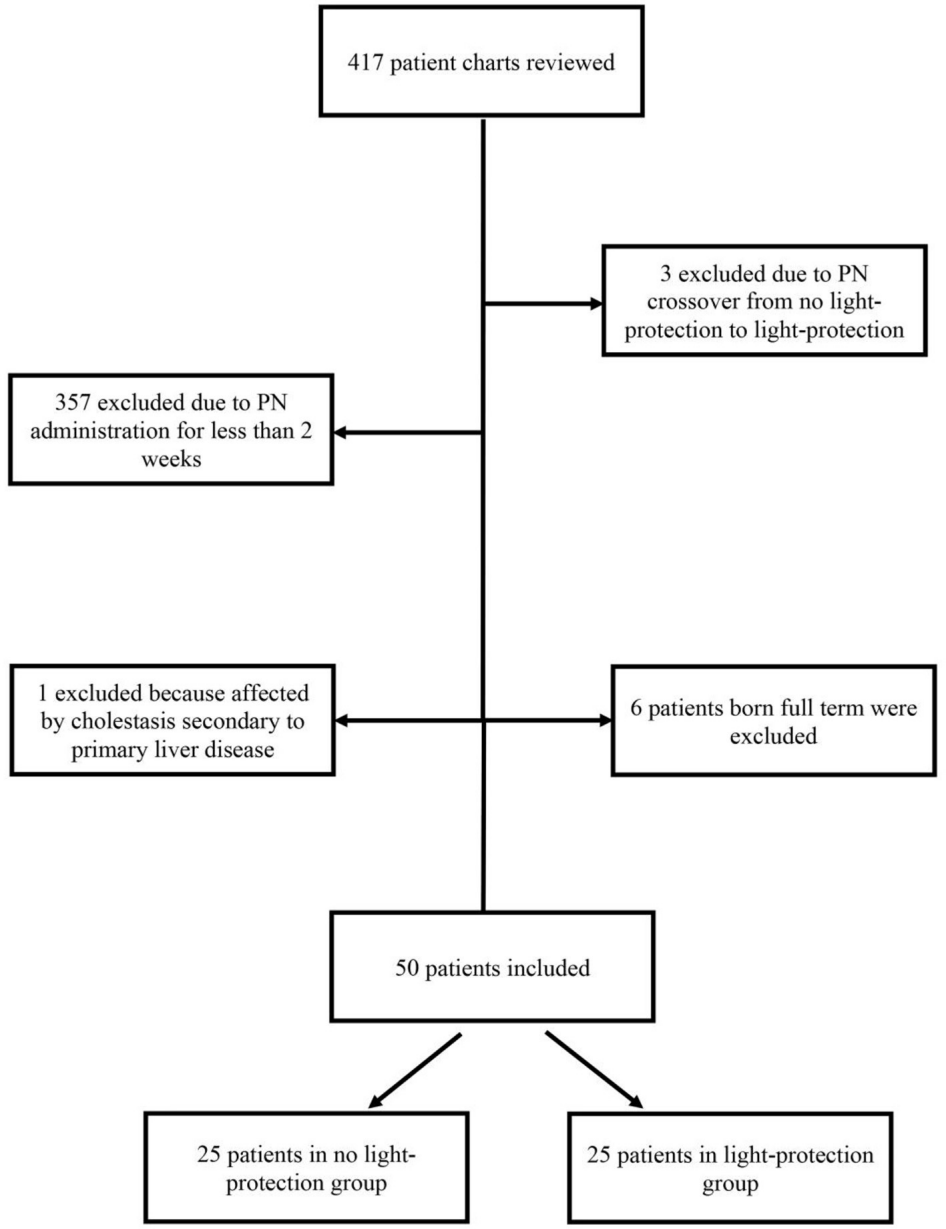
Population flow chart.

Of the 417 patient charts reviewed, 50 infants satisfied the inclusion criteria: 25 patients in the pre-light-protection group and 25 patients in the post-light-protection group (shown in Figure 2). There were no significant differences in terms of sex, birth weight, number of infants in the subcategories of low birth weight (LBW, infant whose birth weight was between 1.5 and 2.5 kg), very low birth weight (VLBW, infants whose birth weight was between 1 and 1.5 kg), extremely low birth weight (ELBW, infants whose birth weight was <1 kg), IUGR status, gestational age, and presence of PDA (Table 1A), and respiratory support at 36 weeks PMA. The direct bilirubin peaked earlier in the light-protected group compared to the no-light-protected group (13.5 vs. 23.7 days, *p* = 0.0087). However, the PMA and the body weight at the time of the direct peak bilirubin did not differ between groups (Table 1A). Among the risk factors for cholestasis, infants in the light-protection group tolerated initiation of enteral feeds earlier compared to infants in the no light-protection group, (3 vs. 6 days, *p* = 0.004; Table 1A) but the median days to reach full enteral feeds in the light-protection group was not statistically different among the two groups (31 days in the no light protection group vs. 25.5 days in the light protection group, *p* = 0.07, Table 1A). Moreover, ANCOVA was used to adjust for DOL of onset of enteral feeding tolerance and of reaching full feeds; and mean peak direct bilirubin levels remained significantly lower in the light-protection group compared to the no light-protection group even after individually adjusting for risk factors (Table 2B).

**Table 1A:** Patient characteristics and primary and secondary outcomes.

**Patient characteristics**			
	**No light-protection** **(*n* = 25)**	**Light-protection** **(*n* = 25)**	***P*-value**
Sex, female (%)	11 (44%)	15 (60%)	0.26
Birth weight (kg)	0.7 (0.5–2.1)	0.8 (0.4–3.7)	0.15
LBW	3 (12%)	2 (8%)	0.1252
VLBW	1 (4%)	6 (24%)	
ELBW	21 (84%)	16 (64%)	
N/A^#^	0 (0%)	1 (4%)	
IUGR status	7 (28%)	4 (16%)	0.30
Gestational age (wks)	26.4 (23.1–33.4)	26.7 (23.9–36.1)	0.30
PDA	11 (44%)	10 (40%)	1.00
**Respiratory support at 36wks PMA**			
Invasive (SIMV/HFJV; %)	3 (12%)	0 (0%)	0.2260
Non-invasive (NIPPV, CPAP, NC; %)	13 (52%)	11 (44%)	
RA	5 (20%)	5 (20%)	
Cycling (CPAP/NC/RA)	2 (8%)	7 (28%)	
N/A	2 (8%)	2 (8%)	
**Patient characteristics at peak direct bilirubin**			
DOL at peak direct bilirubin	23.7 (3-89)	13.5 (3–38)	0.0087
Postmenstrual age (wks) at peak direct bilirubin	29.1 (24.3–38.4)	28.9 (26.1–36.6)	0.34
Weight (kg) at peak direct bilirubin	0.9 (0.5–3.0)	1.0 (0.5–3.8)	0.95
**Risk factors for cholestasis**			
DOL of onset of enteral feeding tolerance	6 (2–26)	3 (1–13)	0.004
DOL when patient reached full feeds	31 (17–99)	25.5 (5–44)	0.07
Duration of PN (days)	27 (14–107)	23 (14–41)	0.06
Lipids intake (g/kg/day)	1.9 (0.6–3.6)	2.2 (1.0–3.2)	0.07
Intralipid	23 (92%)	21 (84%)	0.6671
SMOFlipid	2 (8%)	4 (16%)	
Short bowel syndrome	4 (16%)	1 (4%)	0.35
Phototherapy	25 (100%)	23 (92%)	0.49
Duration of phototherapy (days)	7 (4, 8)	5 (4, 7)	0.3092

Baseline characteristics and respiratory support at 36 wks PMA of the study population were similar between groups. The serum direct bilirubin peaked earlier (day of life [DOL]) in the light protection group, while there was no difference in age or weight between groups at the time of peak direct bilirubin. The DOL of onset of enteral feeding tolerance was earlier in the light protection group compared to the no light protection group; there was no significant difference in other risk factors for cholestasis between groups.

# , of note, in the light protection group, there was one infant who did not fit within the low birth weight categories and, therefore, was labeled at N/A.

PMA, postmenstrual age; LBW, low birth weight; VLBW, very low birth weight; ELBW, extremely low birth weight; N/A, no data; IUGR, intrauterine growth restriction; Wks, weeks; PDA, patent ductus arteriosus; SIMV, synchronized invasive mechanical ventilation; HFJV, high flow jet ventilator; NIPPV, non-invasive positive pressure ventilation; CPAP, continuous positive airway pressure; NC, nasal cannula; RA, room air; DOL, day of life; PN, parenteral nutrition; Intralipid, lipid emulsion (plant-based); SMOFlipid, soy-MCT-olive oil-fish oil lipids/lipid emulsion (fish oil and plant based).

**Table 1B:** Patient characteristics and primary and secondary outcomes.

**Primary outcomes**			
	**No light-protection** **(*n* = 25)**	**Light-protection** **(*n* = 25)**	***P*-value**
Cholestasis	12 (48%)	3 (12%)	0.005
**Laboratory data at DOL 0**			
Direct bilirubin (mg/dL)	0.5 (0.3, 0.7)	0.4 (0.4, 0.6)	0.7899
Total bilirubin (mg/dL)	4 (3.6, 4.6)	4.1 (3.5, 4.7)	0.9690
AST (IU/L) DOL 0	46 (22, 68)	31, (24, 44)	0.3677
ALT (IU/L) DOL 0	10 (4, 15)	6 (5, 9)	0.4952
**Laboratory data at DOL 14**			
Direct bilirubin (mg/dL)	1.2 (0.6, 1.9)	0.7 (0.5, 0.8)	0.007
Total bilirubin (mg/dL)	4.0 (2.5, 5.8)	2.3 (1.6, 3.2)	0.01
AST (IU/L) DOL 14	32.5 (28, 45)	31 (28, 35)	0.71
ALT (IU/L) DOL 14	9.5 (6, 11)	7 (5, 8)	0.08
**Laboratory data at time of peak direct bilirubin**			
Direct bilirubin (mg/dL)	1.7 (0.6–8.9)	0.9 (0.6–3.3)	0.02
Total bilirubin (mg/dL)	4.1 (2.0–11.2)	3.4 (1.3–8.1)	0.05
AST (IU/L)	40 (19–202)	33 (13–98)	0.19
ALT (IU/L)	8 (1–62)	6 (2–80)	0.27
**Treatment of cholestasis**			
Cycling PN	6 (24%)	0 (0%)	0.02
Ursodiol	6 (24%)	1 (4%)	0.09
**Secondary outcomes**			
NEC	10 (40%)	4 (16%)	0.06
Sepsis	7 (28%)	3 (12%)	0.16
ROP	19 (76%)	17 (68%)	0.75
BPD	21 (91%)^*^ out of 23	21 (91%)^*^ out of 23	1.00
Mild	4 (19.1%)	12 (57.1%)	0.0223
Moderate	2 (9.5%)	2 (9.5%)	
Severe	15 (71.4%)	7 (33.3%)	
Death	2 (8%)	1 (4%)	1.00

The rate of cholestasis was significantly less in the light protection group compared to the no light-protection group. Direct bilirubin levels were significantly lower in the light protection group at DOL 14 and at the time of peak direct bilirubin. There was no statistically significant difference between groups for the secondary outcomes. An equal number of patients developed BPD; however, there was a trend of a lower rate of severe BPD after light protection.

* , BPD rate was based on n = 23 patients in both the groups. mg/dL, milligrams per deciliter; IU/L, international units per liter; AST, aspartate aminotransferase; ALT, alanine aminotransferase; DOL, day of Life; PN, parenteral nutrition; NEC, necrotizing enterocolitis; ROP, retinopathy of prematurity; BPD, bronchopulmonary dysplasia.

The light protection group had a shorter duration of PN; however, although there was a trend, the difference did not reach statistical significance (27 vs. 23 days, *p* = 0.06). The lipid intake (g/kg/day) and type of lipid did not differ between the two groups (Table 1A). Of note, two patients in the light protection group were switched from Intralipid^®^ to SMOFlipid^®^ during the study; however, both of these infants received Intralipid^®^ at the time of peak serum direct bilirubin and therefore were recorded as receiving Intralipid^®^ as this was the time of the primary outcome. The use of phototherapy and its duration were similar between the two groups (*n* = 25 in the no light protection, *n* = 23 in the light protection groups), *p* = 0.49 (Table 1A).

### PN light-protection and primary outcomes

**Figure 3:**
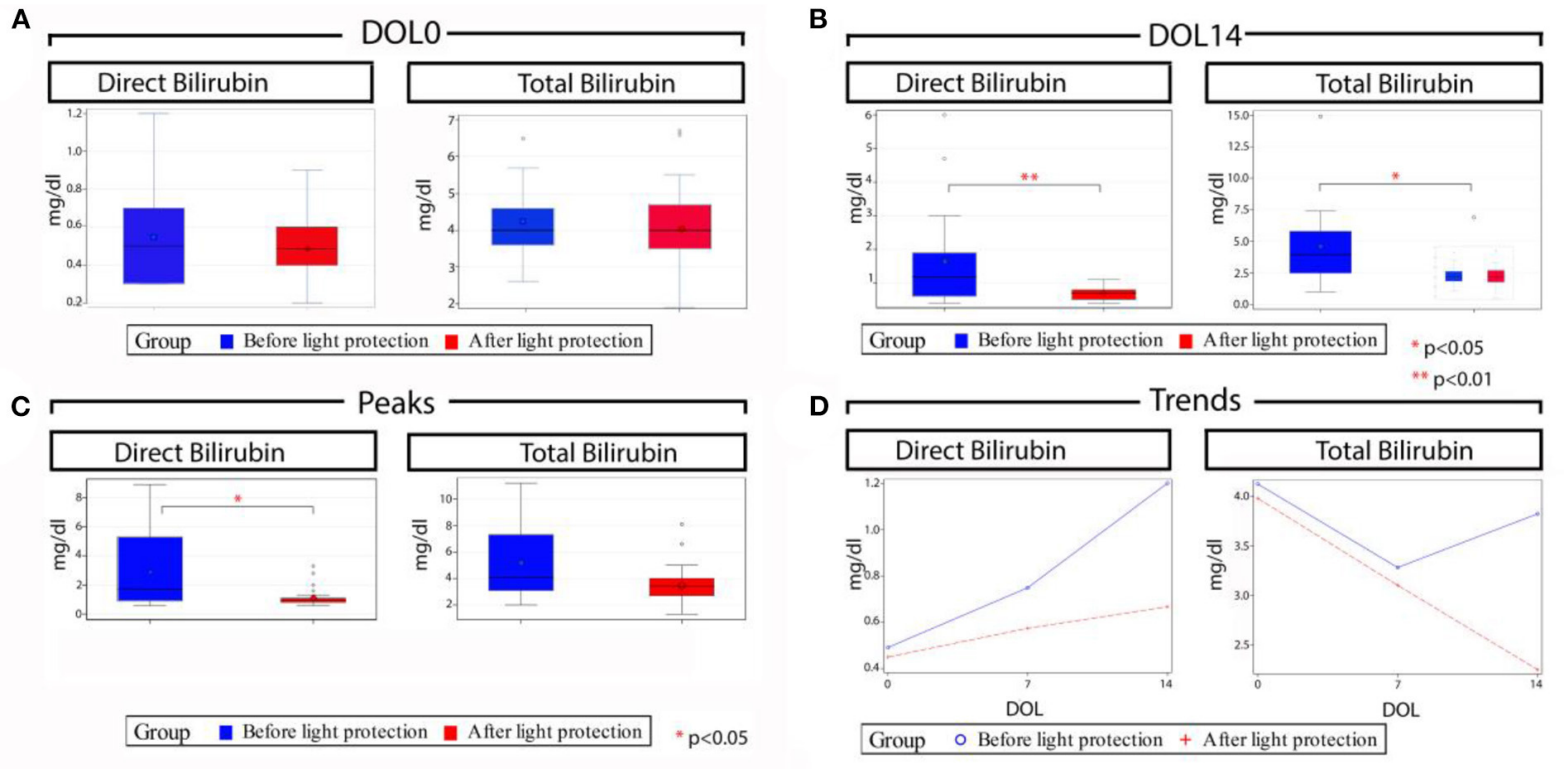
Total and direct bilirubin levels before and after light protection at different time points. **(A)** Median direct and total bilirubin levels at day of life (DOL) 0 were similar in both groups; **(B)** Median direct and total bilirubin levels at DOL 14 were significantly lower in the light protection group compared to the no light protection group; **(C)** Median peak direct bilirubin was significantly lower in the light protection group with no significant difference in median peak total bilirubin levels; and **(D)** Serum direct and total bilirubin trends across time in the two groups; of note bilirubin trends in both groups started to differentiate after 7 days of PN. DOL, Day of life.

The primary outcome, the incidence of cholestasis, was significantly decreased in the light protection group, (3 vs. 12, *p* = 0.005; Table 1B). At birth, total and direct bilirubin levels as well as AST and ALT levels were not different between the two groups (Table 1B; Figure 3A). However, at 14 DOL, both direct and total bilirubin levels were significantly reduced in the light-protected group (shown in Table 1B; Figure 3B). At the time of peak serum direct bilirubin, the median direct bilirubin in the no-light-protection group was 1.7 mg/dl [0.6–8.9] compared to 0.9 mg/dl [0.6–3.3] in the light-protection group, *p* = 0.02 (Table 1B; Figure 3C). The median peak total bilirubin was not different between the no-light-protection group (4.1 mg/dl [2.0–11.2]) and the light-protected group (3.4 mg/dl [1.3–8.1]), *p* = 0.05 (Table 1B; Figure 3C). Both the direct and total bilirubin's trends started to differentiate after 7 days of PN (Figure 3D). There was no difference in AST and ALT levels between the two groups at DOL 0, DOL 14, and at the time of peak direct bilirubin (Table 1B). There were no patients in the light-protection group who required cycling PN (six vs. zero patients), *p* = 0.02 (Table 1B). The use of ursodiol was not statistically significant between the two groups, six vs. one, *p* = 0.09 (Table 1B).

### PN light-protection and secondary outcomes

The impact of light protection of PN on clinical outcomes was recorded as secondary outcomes.

Necrotizing enterocolitis occurred in 10 infants in the no light-protection group and four infants in the light-protection group, *p* = 0.06 (Table 1B). In each group, two patients were excluded from BPD evaluation due to either death or transfer to another facility prior to 36 weeks of PMA. An equal number of patients developed BPD (21/23 in each group), *p* = 1.00 (Table 1B). Reviewing the different severities of BPD, there was a decrease in severe BPD (7 vs. 15) and an increase in mild BPD (12 vs. 4) after light protection, *p* = 0.0223. Furthermore, the rate of sepsis, PDA, ROP, and death did not differ between the two groups (Table 1B).

The PN light exposure was independently associated with cholestasis in the univariate analysis; other variables associated with cholestasis were DOL when the infant reached full feeds, PN duration, and presence of NEC (Table 2A).

**Table 2A:** Univariate analyses and ANCOVA analyses.

	**Univariate analyses**				
**Variables**	**Reference group**	**Unadjusted odds ratio**	**95% Interval confidence**		***p*-value**
Exposure to light	*Before vs. after light protection*	6.77	1.60	28.53	0.0092
Sex	*Female vs. male*	1.08	0.32	3.63	0.9017
Birth weight	*Unit=1*	0.08	0.004	1.32	0.0773
IUGR status	*Presence vs. absence*	1.45	0.35	5.97	0.6031
Gestational age	*Unit=1*	0.88	0.69	1.13	0.3212
DOL of onset of enteral feeding tolerance	*Unit=1*	1.05	0.95	1.17	0.3097
DOL of reaching full enteral feeds	*Unit=1*	1.22	1.07	1.40	0.0034
Duration of PN	*Unit=1*	1.20	1.08	1.34	0.0009
Lipid type	*Intralipid vs. SMOFlipid*	0.375	0.066	2.121	0.2672
SBS	*Presence vs. absence*	12.36	1.28	122.62	0.0317
NEC	*Presence vs. absence*	9.00	2.22	36.54	0.0021
Sepsis	*Presence vs. absence*	1.76	0.41	7.44	0.4436
Duration of phototherapy	*Presence vs. absence*	1.08	0.92	1.27	0.3352

Univariate logistic regression model for predicting cholestasis was performed. Parenteral nutrition light exposure was independently associated with cholestasis. The day of life when the patient reached full feeds, duration of PN, SBS, and NEC were also associated with cholestasis.

IUGR, Intrauterine growth restriction, DOL, day of Life; PN, parenteral nutrition; SBS, short bowel syndrome; NEC, necrotizing enterocolitis.

Differences in mean peak direct bilirubin levels were adjusted for birth weight, duration of PN, DOL of onset of enteral feeding tolerance and of reaching full feeds, presence of SBS and NEC, type of lipid (SMOFlipid^®^ vs. Intralipid^®^), phototherapy treatment and its duration using ANCOVA and remained significantly lower in the light-protection group compared to the no light-protection group even after individually adjusting for risk factors (Table 2B).

**Table 2B:** Univariate analyses and ANCOVA analyses.

**ANCOVA analyses**					
**Covariate**	**Group**	**Geometric mean**	**Lower confidence limit**	**Upper confidence limit**	***p-*value**
Birth weight (kg)	*No LP*	1.93	1.46	2.55	0.0029
	*LP*	1.06	0.81	1.40	
Duration of PN	*No LP*	1.71	1.38	2.13	0.0283
	*LP*	1.20	0.97	1.49	
DOL of onset of enteral feeding tolerance	*No LP*	1.95	1.45	2.61	0.0060
	*LP*	1.06	0.79	1.41	
DOL of reaching full enteral feeds	*No LP*	1.55	1.25	1.90	0.0360
	*LP*	1.12	0.91	1.38	
Presence of SBS	*No LP*	2.80	2.02	3.88	0.0062
	*LP*	1.66	1.14	2.42	
Presence of NEC	*No LP*	2.13	1.66	2.74	0.0138
	*LP*	1.35	1.02	1.79	
Lipid type (SMOFlipid^®^ vs. Intralipid^®^)	*No LP*	2.50	1.72	3.61	0.0008
	*LP*	1.25	0.89	1.76	
Phototherapy	*No LP*	2.05	0.94	4.47	0.0025
	*LP*	1.06	0.51	2.20	
Duration of phototherapy (days)	*No LP*	1.95	1.46	2.59	0.0062
	*LP*	1.04	0.77	1.41	

ANCOVA models were run separately for each covariant to determine if there was a difference between the groups regarding direct bilirubin level. Mean peak direct bilirubin levels remained significantly lower in the light-protection group compared to the no light-protection group even after individually adjusting for risk factors.

LP, light protection; PN, parenteral nutrition; DOL, day of Life; SBS, short bowel syndrome; NEC, necrotizing enterocolitis; SMOFlipid, soy-MCT-olive oil-fish oil lipids/lipid emulsion (fish oil and plant based); Intralipid, lipid emulsion (plant-based).

## Discussion

In this single-center retrospective study, we found a significant reduction in the incidence of cholestasis and peak serum direct bilirubin with the use of light-protected PN. Our data support that shielding PN from light may be associated with improved hepatobiliary markers such as direct bilirubin. These data support the European Medicines Agency and ASPEN's recommendation to light protect PN (10, 11).

To our knowledge, this is the first study that suggests a reduced incidence of IFALD with light protection of PN in premature infants *in vivo*. Contrary to our findings, Laborie et al. published a study of 587 infants which found light protection of PN had no effect on cholestasis. However, in this study, the primary endpoint was the effect of light-protected PN on BPD or death at 28 DOL and not the presence of IFALD. Moreover, the authors only evaluated the serum direct bilirubin in patients after cholestasis developed, while we looked at both separately, the incidence of cholestasis and the direct bilirubin levels (6).

Numerous investigations report the hepatobiliary effects of protecting PN from light in animal models, such as guinea pigs that, along with primates, are the only animal species that cannot synthesize vitamin C. Moreover, newborn guinea pigs have an immature glutathione synthesis making them similar to newborn humans (13, 14). In animal models, light-exposed PN was found to decrease bile flow and increase hepatic necrosis, portal tract inflammation, and biliary concentrations of oxidized glutathione (antioxidant produced primarily by the liver) (15).

These data supported the hypothesis that light-exposed PN can cause hepatic damage in animal models. Autopsy reports of 24 human newborns who received PN confirmed the presence of a progression in the severity of hepatic histopathological changes in relation to the duration of PN administration (3).

Photosensitive PN components, which are considered responsible for cellular damage after light exposure, include multivitamins, lipid emulsions, and amino acids. Vitamins are the main sources of peroxides in PN solutions; riboflavin and ascorbate are the most claimed vitamins attributed to the process of peroxide generation (16). Laborie et al. identified vitamins as the major origin of oxidative stress and light protection of PN as a strategy to reduce it (17). Lipid emulsions can increase the susceptibility for peroxide formation, particularly pure soy-bean-based emulsions such as Intralipid^®^ 20% (Fresenius Kabi) (16). The use of SMOFlipid^®^ 20% (Fresenius Kabi) may help decrease the risk of developing cholestasis as it does not contain a high percentage of phytosterols compared to Intralipid^®^ (18). In addition, SMOFlipid^®^ is hypothesized to reduce oxidative stress due to a decreased level of omega-6 fatty acids (pro-inflammatory substance) compared to Intralipid^®^ soybean oil. Moreover, SMOFlipid^®^ contains non-inflammatory omega-3 fatty acids (DHA and EPA) and approximately 200 mcg/ml of anti-oxidant alpha-tocopherol (vitamin E) (19). In our study, we found that even with an increased (although not statistically significant) daily dose of lipids in the light-protected group (2.2 vs. 1.9 mg/kg/day, *p* = 0.07); the direct bilirubin levels were statistically significantly lower in this group of patients compared to the no-light-protected group. In addition, the mean peak direct bilirubin levels remained significantly lower in the light-protection group compared to the no light-protection group even when adjusted for lipid type (SMOFlipid^®^ vs. Intralipid^®^) using ANCOVA.

A clinical study performed on preterm infants reported that light protection of PN was associated with an increase in the tolerance of enteral intake during the first week of life (20). Shielding PN from light decreases the generation of vasoactive oxidants, which can cause vasoconstriction and may affect tolerance to enteral feedings due to an improved splanchnic flow (21). Our data support these findings, showing that patients tolerated earlier initiation of enteral feeds with PN light protection and an early reach of full feeds. The earlier and better tolerance of enteral feeding could explain the decreased trend of liver injury markers (ALT/AST, although the trend was not statistically significant) and cholestasis incidence in the light-protected PN group. However, our univariate analysis did not support these findings, since the light protection group patients had a statistically significant earlier onset of enteral feeding, as well as reaching full feeds (although not statistically significant); further studies would be helpful to investigate and confirm these findings. The prevention of vasoconstriction of the splanchnic vasculature can result in reduced incidence of NEC, which can also have a major effect on tolerance of enteral feedings. Premature infants who develop NEC, in fact, are at higher risk for IFALD due to their liver immaturity, as well as exposure to sepsis, intestinal obstruction, development of SBS, withholding of enteral feeds, and increased content of lipid and glucose in PN to meet energy needs (8).

Finally, NEC itself is a risk factor for the development of IFALD (22, 23). Indeed, NEC alters hepatobiliary function causing biliary stasis and mild hepatocyte degeneration, leading to increased susceptibility to hepatic injury (23). In our study, 40% of the infants in the no light protection group developed NEC compared to 16% in the light protection group. Because the light protection of the PN was started at birth and NEC developed later in life, it is possible to hypothesize that light protection may decrease the risk of the development of NEC because of a decrease in oxidative stress. However, because damage from NEC increases susceptibility to hepatic damage and infants affected by NEC are usually on PN, it is very difficult to separate these two causes as the reason for cholestasis. Further studies are necessary to evaluate whether light-protected PN has an effect on NEC-induced cholestasis. Finally, surgical NEC, considered the most severe form of this disease, can lead to SBS, defined as the spectrum of malabsorption that occurs after resection of a major portion of the small intestine. The previous data showed that cholestasis and liver failure occurs in a greater proportion of patients with SBS probably secondary to a longer duration of PN requirement and an increased number of septic complications and malnutrition (24). In our population, there was an increased number of patients with SBS in the no-light-protection group compared to the infants with light-protected PN; this difference, however, was not statistically significant. As expected, in the univariate analysis, the presence of both NEC and SBS was associated with cholestasis; when the mean peak direct bilirubin levels were adjusted for NEC and SBS using ANCOVA, direct bilirubin levels remained significantly lower in the light-protection group compared to the no light-protection group. These data support our conclusion that light protection is associated with a decreased incidence of IFALD and peak direct bilirubin.

A randomized control study did not show a decrease in the rate of BPD when PN was light-protected (6). Similarly, in our retrospective analysis, the overall incidence of BPD was the same in the two groups. There was, however, a shift from severe BPD to mild BPD after the introduction of PN light protection. Due to the retrospective nature of our study, a small patient cohort, and the innate multifactorial pathogenesis of BPD, further studies would be beneficial to support our findings that light protection may decrease the risk of severe BPD. A *post-hoc* analysis published in 2007 showed a 30% decrease in the rate of BPD when PN was completely (bags and tubing) photo-protected compared with partial photoprotection (bags only) (25). The data presented in our work were obtained shielding only the bags and this may explain the lack of effect on the overall rate of BPD or other diseases connected with increased oxidant stress. Another explanation may be related to the multifactorial pathogenesis of BPD that is associated not only with oxidative stress and therefore with secondary inflammation, but also with other factors such as lung immaturity and infection.

Despite light-exposed PN solutions being cytotoxic *in vitro*, their peroxide content poses bacteriostatic properties *in vitro* (26). Hence, light protection might enhance the risk of late-onset sepsis in this vulnerable population. In our population, photoprotection was not associated with an increased rate of sepsis, supporting the safety and the benefits of light-protecting PN (15).

Limitations to our study include the retrospective nature of the design. There was incomplete light protection during the compounding of the PN and the tubing of the PN remained exposed to light. We did not calculate a formal sample size; this was a small convenience sample based on feasibility (*n* = 50). We performed a statistical analysis once we reached 25 patients in the light protection group and due to the positive impact observed with light protection of the PN bags, the decision was made to stop this study and initiate complete light protection. Our study team is in the process of conducting a larger study that provides light protection for the PN bags, lipids, and tubing. We acknowledge that measurements of oxidative stress would be important to evaluate the efficacy of these interventions and we are, therefore, planning to measure oxidative stress using urine samples from infants with and without completely light-protected PN in our next study.

## Conclusion

Light-protecting PN preparations were associated with a decreased incidence of IFALD and peak direct bilirubin. Moreover, there was a trend toward decreased incidence of severe BPD in the light-protection group. Further prospective controlled studies are needed to confirm these findings.

## Data availability statement

The raw data supporting the conclusions of this article will be made available by the authors, without undue reservation.

## Ethics statement

The studies involving human participants were reviewed and approved by NYU Langone Hospital–Long Island Institutional Review Board, approval number 1325821-1. Written informed consent from the participants' legal guardian/next of kin was not required to participate in this study in accordance with the national legislation and the institutional requirements.

## Author contributions

CC, MM, and CT conceptualized and designed the study, did acquisition of data, helped in analysis and interpretation of data, drafted the initial manuscript, and critically reviewed and revised the manuscript. VT and MA provided substantial contribution to acquisition of data and critically reviewed and revised the manuscript. GE-C and NH contributed toward conceptualization, designed the study, and served as investigators in the study. All authors contributed to the article and approved the submitted version.

## Conflict of interset

The authors declare that the research was conducted in the absence of any commercial or financial relationships that could be construed as a potential conflict of interest.

## Publisher's note

All claims expressed in this article are solely those of the authors and do not necessarily represent those of their affiliated organizations, or those of the publisher, the editors and the reviewers. Any product that may be evaluated in this article, or claim that may be made by its manufacturer, is not guaranteed or endorsed by the publisher.
